# Analysis of the spatial-temporal distribution characteristics of hepatitis E in Jiangsu province from 2005 to 2020

**DOI:** 10.3389/fpubh.2023.1225261

**Published:** 2023-08-08

**Authors:** Yao Shi, Wenqi Shen, Wendong Liu, Xuefeng Zhang, Qingxiang Shang, Xiaoqing Cheng, Changjun Bao

**Affiliations:** ^1^Taicang City Centre for Disease Control and Prevention, Suzhou, Jiangsu, China; ^2^Jiangsu Field Epidemiology Training Program, Jiangsu Provincial Centre for Disease Control and Prevention, Nanjing, Jiangsu, China; ^3^Jiangsu Provincial Centre for Disease Control and Prevention, Jiangsu Institution of Public Health, Nanjing, Jiangsu, China; ^4^School of Public Health, Nanjing Medical University, Nanjing, Jiangsu, China

**Keywords:** hepatitis E (HE), spatial-temporal distribution, space-time scanning, epidemiology characteristic, clustering

## Abstract

**Objective:**

This study attempts to analyze the spatial clustering and spatial-temporal distribution characteristics of hepatitis E (HE) at the county (city and district) level in Jiangsu province to provide a scientific basis for the prevention and control of HE.

**Method:**

The information on HE cases reported in the Chinese Center for Disease Control and Prevention Information System from 2005 to 2020 was collected for spatial autocorrelation analysis and spatial-temporal clustering analysis.

**Result:**

From 2005 to 2020, 48,456 HE cases were reported in Jiangsu province, with an average annual incidence rate of 3.87/100,000. Male cases outnumbered female cases (2.46:1), and the incidence was highest in the 30–70 years of age group (80.50%). Farmers accounted for more than half of all cases (59.86%), and in terms of the average annual incidence, the top three cities were all in Zhenjiang city. Spatial autocorrelation analysis showed that Global Moran's I of HE incidence varied from 0.232 to 0.513 for the years. From 2005 to 2020, 31 counties (cities and districts) had high and statistically significant HE incidence, and two clustering areas were detected by spatial-temporal scanning.

**Conclusion:**

HE incidence in Jiangsu province from 2005 to 2020 was stable, with age and gender differences, regional clustering, and spatial-temporal clustering. Further investigation of HE clustering areas is necessary to formulate corresponding targeted prevention and control measures.

## 1. Introduction

Hepatitis E virus (HEV) is a non-enveloped, positive-strand, single-stranded RNA virus with a diameter of 27–30 nm. The majority of concerning HEV genotypes belong to the *Paslahepevirus* genus and are subsequently divided into eight genotypes. HEV genotypes 1 and 2 exclusively infect humans and primates while genotypes 3 and 4 infect both humans and other mammals. However, HEV genotypes 5 and 6 are isolated from wild boars, and genotypes 7 and 8 were identified from camels in the United Arab Emirates and China, respectively (1, 2). HE, acute viral hepatitis with liver inflammation and necrosis as the main pathological changes, is a category B infectious disease in China. The main transmission routes include drinking water contaminated with HEV, blood transfusion, and consumption of undercooked animal products (3).

HEV infection usually presents as self-limiting, but some patients may develop acute hepatitis or acute liver failure, with symptoms such as jaundice, loss of appetite, aversion to oil, fatigue, fever, and somnolence (3). Immunocompromised populations such as organ transplant recipients, patients with blood system diseases, and pregnant women are more susceptible to chronic HEV infection, which may lead to liver cirrhosis (4). Moreover, a meta-analysis of HEV infection during pregnancy showed a symptomatic HEV infection rate of 49.6% (5). HEV infection in pregnant women poses serious risks to both the mother and fetus, increasing the rates of premature birth and stillbirth, with fetal and neonatal mortality rates of 33 and 8%, respectively (6).

Currently, HEV is divided into eight genotypes. Genotypes 1–4 mainly infect humans. Specifically, genotypes 1 and 2 only infect humans, while genotypes 3 and 4 can infect both humans and pigs (7). Genotypes 1 and 2 are mainly prevalent in underdeveloped countries with poor sanitation, and the outbreaks or epidemics are largely caused by water sources contaminated with feces (8). Genotype 3 is mainly prevalent in developed countries, while genotype 4 is mainly prevalent in China and Southeast Asia, usually in sporadic cases (9).

Globally, according to WHO estimates, there are ~20 million new cases of HEV infection each year, of which 3.3 million develop hepatitis symptoms. In 2015, ~44,000 HEV infection cases died worldwide, accounting for 3.3% of deaths from viral hepatitis (10). Similarly, seroepidemiologic studies have shown that approximately one-third of the global population has been infected with HEV (11).

Regarding issues in China, HE has been reported since 1982. From 1986 to 1988, one of the largest HE epidemics in the world occurred in Xinjiang, with ~120,000 cases of infection and over 700 deaths (12). In recent years, the reported incidence of HE has shown an increasing trend. Particularly since 2012, the reported number of HE cases has exceeded that of hepatitis A for 9 consecutive years, and HE has become one of the main types of acute viral hepatitis in adults in China (13). A meta-analysis of primary risk factors of HEV infection showed that 44–83% of patients with chronic hepatic disease were at risk of HEV superinfection, with a mortality rate of up to 75% (14).

Jiangsu is one of the regions with a high prevalence of HE in China, and HEV infection imposes a heavy economic burden on families and society. Cui et al. found that inpatients with HE spent more than half of their per capita disposable income on the disease, suggesting that HE had become a public health concern in China and around the world (15).

HEV has a broad host range and geographical distribution (16). As transportation rapidly develops, cross-regional supply of non-prepackaged products has become common. In certain cases, hog trade is regarded as the main reason for HEV transmission (17). The geographical characteristics of HE incidence are gradually becoming obvious, and spatial analysis methods can more directly reveal the geographical distribution status of the disease and discover its latent spatial clustering phenomenon and spatial differences, further identifying high-risk areas and populations (18).

Currently, the application of spatial analysis methods to explore the spatial-temporal clustering of various types of enteric infectious diseases has become a research hotspot. According to the literature, however, there is a lack of analysis of the prevalence and spatial-temporal clustering of HE within Jiangsu province, although it is a hotspot for HE incidence in China (19).

In this study, a descriptive analysis of disease distribution and spatial clustering was conducted on the number of HE cases reported in Jiangsu province from 2005 to 2020 to investigate the epidemiological trends and distribution characteristics of HE in the province, in order to explore the key prevention and control seasons, populations, and regions and analyze and guide the formulation of subsequent prevention and control strategies and measures.

## 2. Materials and methods

### 2.1. Study area

Jiangsu province is located in the Yangtze River Delta region between geographical coordinates of 116°21′ and 121°56′ E longitude and 30°45′ and 35°08′ N latitude. This province, with Nanjing as its capital, has 13 municipal-level administrative divisions and 95 county-level administrative divisions. The coastal province boasts lakes and has flat terrain, and its landscape consists of plains, waters, and low hills. It is bordered by the Yellow Sea to the east and straddles two water systems, the Yangtze River and the Huai River. The resident population of the province was 85.15 million at the end of 2022, with a total regional GDP of 12,287.56 billion yuan. At the end of 2022, the province had 85.15 million permanent residents, and its regional GDP reached 12,287.56 billion yuan.

### 2.2. Data source

The individual case information of reported HE cases in Jiangsu province from 1 January 2005 to 31 December 2020, with current addresses of patients in Jiangsu province, was extracted from the Chinese Center for Disease Control and Prevention Information System. Demographic data for the same period were obtained from the Basic Information System of the Chinese Center for Disease Control and Prevention Information System.

### 2.3. Methods

The distribution characteristics of reported HE cases in Jiangsu province from 2005 to 2020 were analyzed using descriptive epidemiological methods. In order to make it more intuitive, ArcGis10.3 software was used to plot maps and visualize them.

#### 2.3.1. Spatial autocorrelation analysis

Spatial autocorrelation analysis, including global spatial autocorrelation and local spatial autocorrelation, was conducted using GeoDa 1.18 software for the incidence of HE in each year from 2005 to 2020, with counties (districts and cities) as the basic units.

Global spatial autocorrelation is used to determine whether there is overall spatial clustering, represented by Global Moran's I with a [−1, 1] range, which is used to indicate the degree of spatial correlation. When I > 0, there is a positive spatial association. The closer the value is to 1, the more similar the spatial associations and properties are, indicating clustering. When I < 0, there is a negative spatial association. The closer the value is to −1, the greater the differences between the research units are, indicating a dispersed distribution. When I = 0, there is no spatial association, and the research objects are randomly distributed (20, 21).

Moreover, when significant global spatial autocorrelation changes exist in the study area, local spatial autocorrelation analysis is conducted using local indicators of spatial association (LISA) to identify and explore hot spots and cold spots of the disease incidence in the space (22). In this study, local spatial autocorrelation analysis was conducted at the county (district or municipal) level. LISA is classified into five categories: High-High (H-H), Low-Low (L-L), None (spatial outliers not significant), High-Low (H-L), and Low-High (L-H) (20, 21). *Z*-tests were used to assess the significance of the statistics at a level of α = 0.05.

#### 2.3.2. Spatial-temporal clustering analysis

SaTScan10.0 was used for spatial-temporal scanning analysis to detect spatial clustering and clustering areas. The principle of this method is to establish cylindrical scanning windows with dynamic changes in size and position. Based on the actual number of cases and population in each scanning window, the theoretical number of cases can be calculated using the discrete Poisson model. The actual number of cases and the theoretical number of cases inside and outside a scanning window are used to construct the log-likelihood ratio (LLR) for the test statistics and evaluate whether the number of cases within the window is abnormal. The Monte Carlo randomization is used to obtain the *P*-value (23). The areas with the highest and statistically significant values of LLR were defined as primary clustering areas, and other areas with statistically significant LLR were defined as secondary clustering areas (8).

In this study, the spatial units were set as counties (cities and districts), and the basic time unit was a month. HE cases reported in the 95 counties (cities and districts) of Jiangsu province from January 2005 to December 2020 were scanned. The maximum risk population was set to 50% of the total population, and the maximum time scanning window was set to 1 month. The model was set to “Poisson,” Monte Carlo simulation was performed 999 times at a level of α = 0.05.

## 3. Results

### 3.1. Time distribution

**Figure 1:**
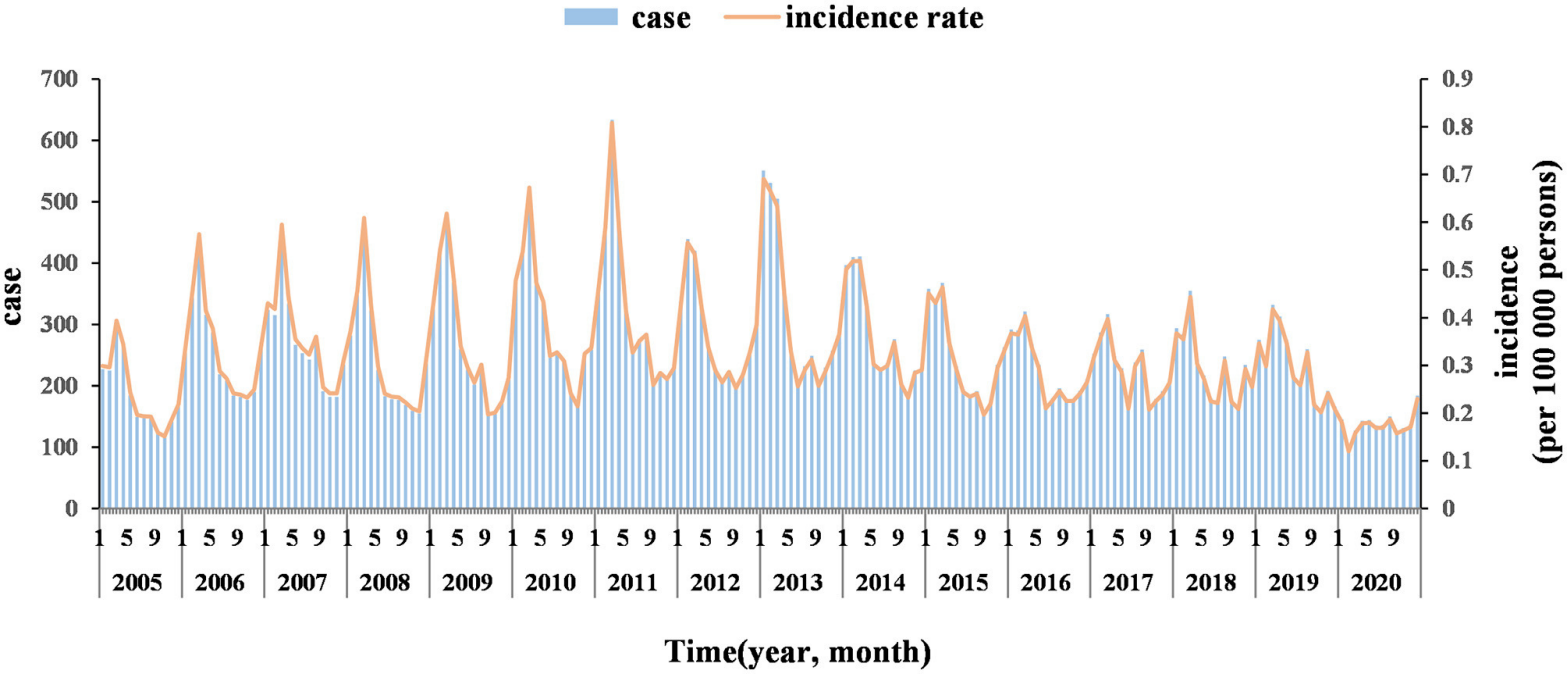
Number of HE cases and the incidence reported in Jiangsu province from 2005 to 2020.

**Figure 2:**
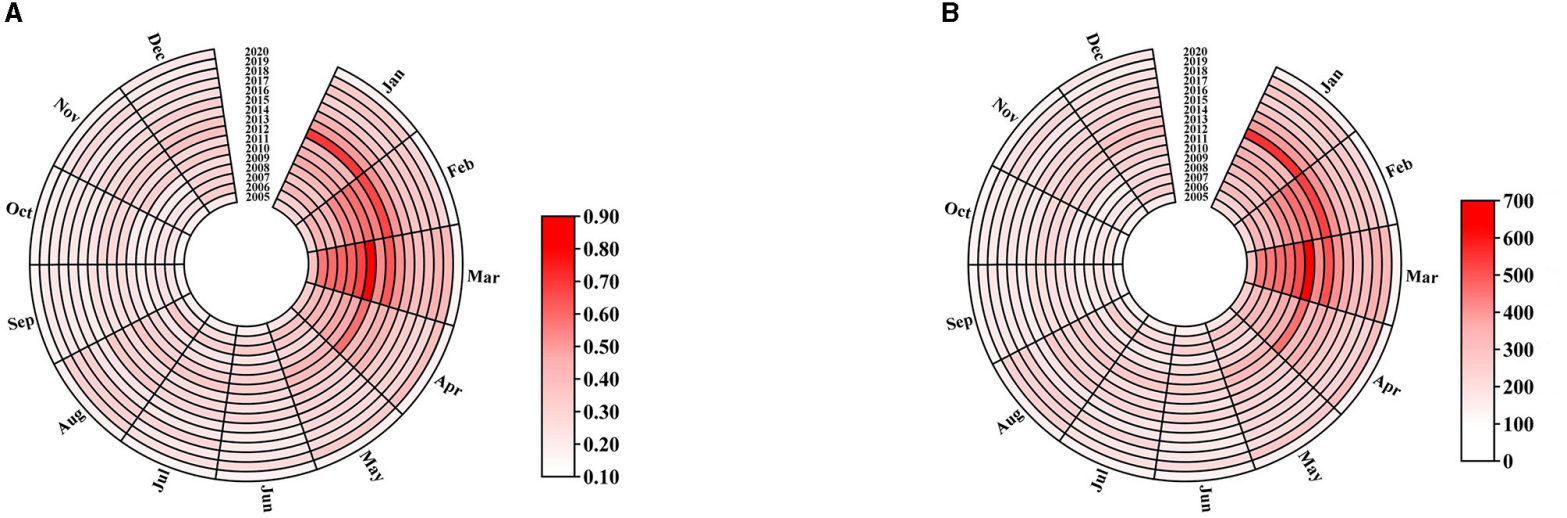
Monthly distribution of the incidence **(A)** and the number of HE cases **(B)** reported in Jiangsu province from 2005 to 2020.

From 2005 to 2020, 48,456 HE cases were reported in Jiangsu province. The number of HE cases and the incidence showed an upward trend from 2005 to 2011, followed by a fluctuating downward trend, and both reached their lowest point in 2020 (Figure 1). In addition, HE cases were reported monthly between 2005 and 2020, and the number of cases showed an overall unimodal distribution with a clear seasonal pattern. The peak incidence occurred in spring, accounting for 34.92% of all cases, followed by summer, which accounted for 25.14% of the total. The month with the most reported cases was March, accounting for 13.27% of the total, while that with the least reported cases was September, accounting for 5.76% of all cases (Figure 2).

### 3.2. Population distribution

**Figure 3:**
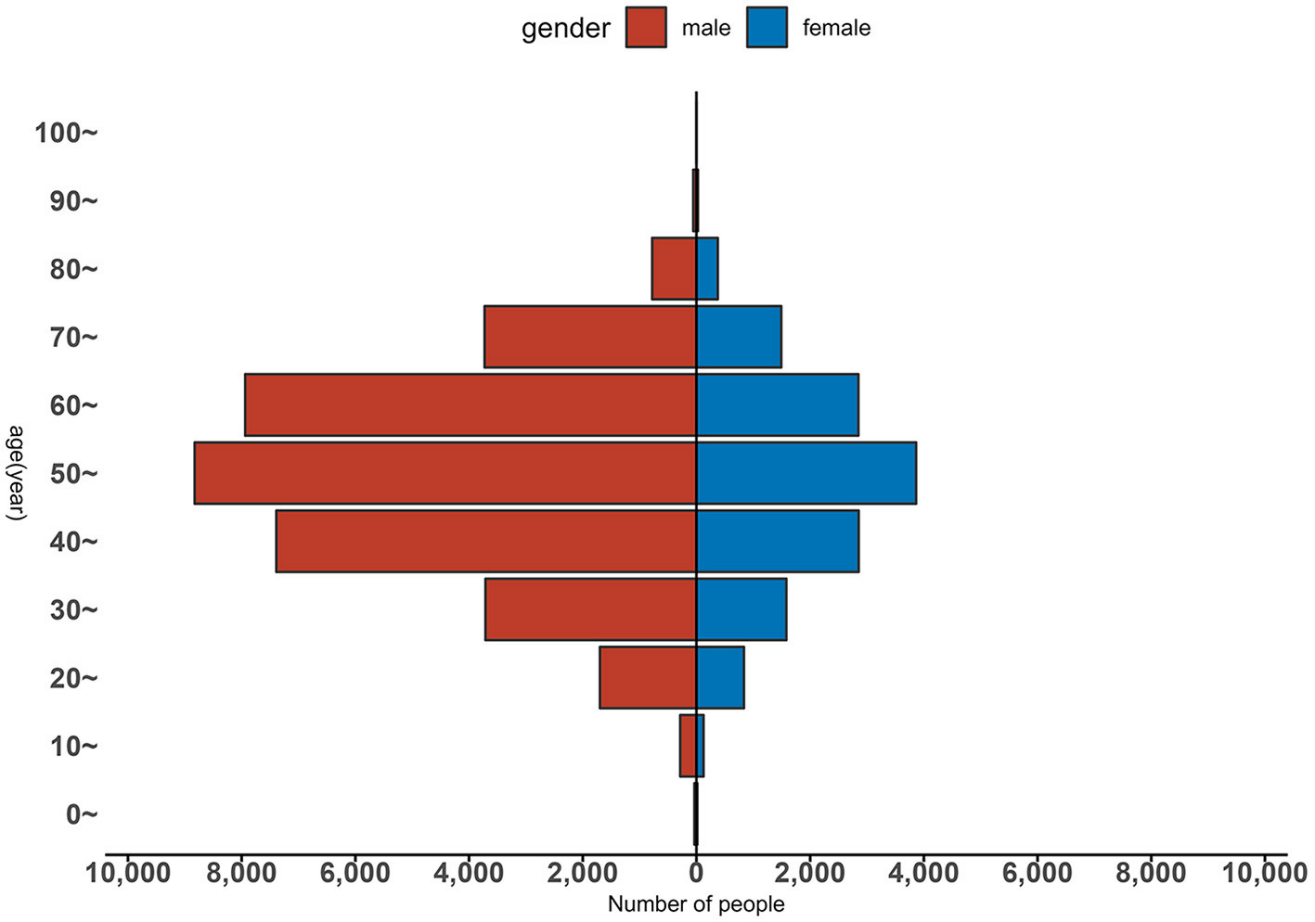
Gender and age distribution of HE patients in Jiangsu province from 2005 to 2020.

**Figure 4:**
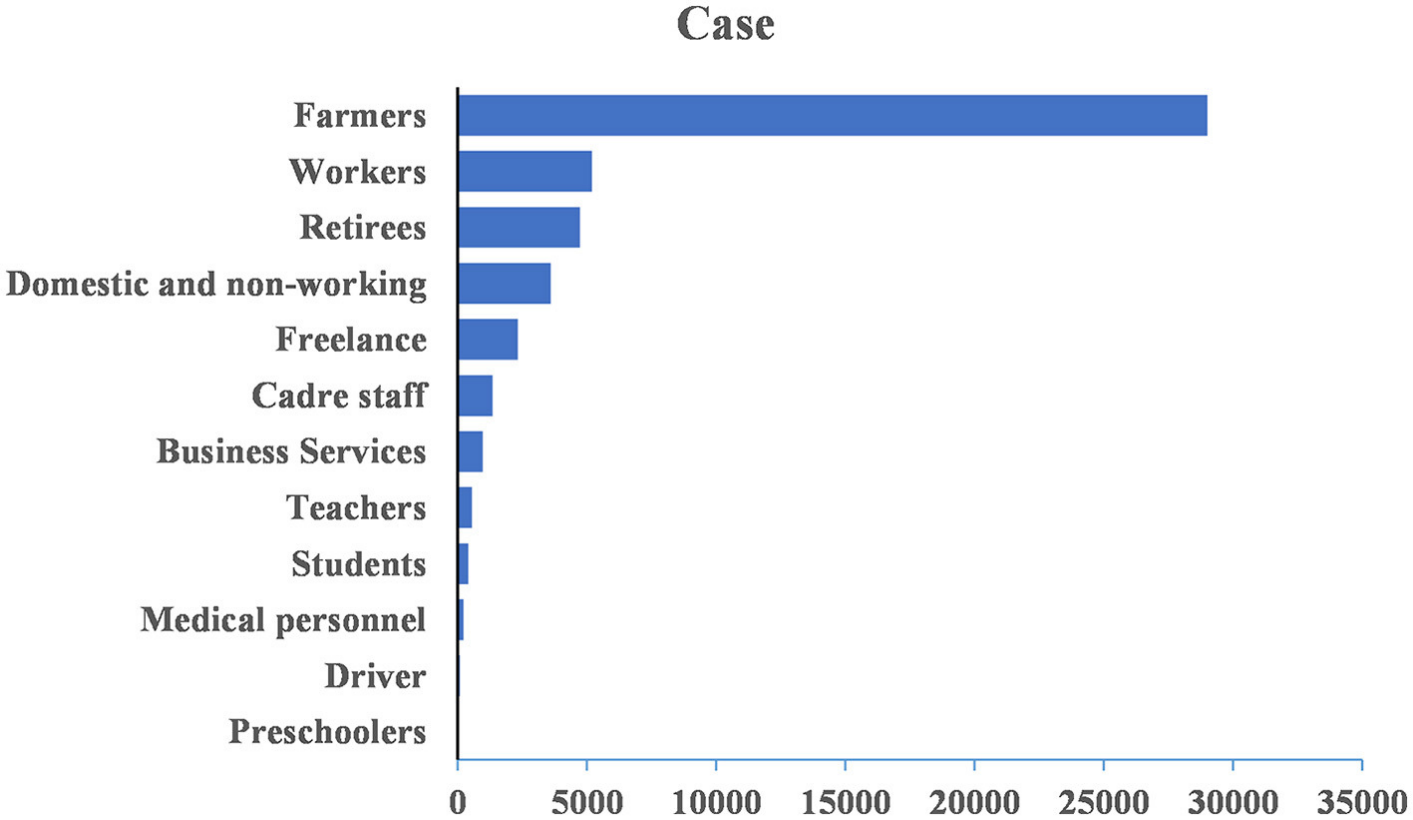
Occupation distribution of HE cases reported in Jiangsu province from 2005 to 2020.

From 2005 to 2020, of all cases, 34,445 were males and 14,011 were females, with a male-to-female ratio of 2.46:1. Cases were reported in all age groups, with the majority distributed between 30 and 70 years old (39,010 cases, 80.50%), followed by those older than 70 years (6,453 cases, 13.32%) and those younger than 30 years (2,993 cases, 6.18%; Figure 3). Additionally, among the 48,456 cases, the top three occupations were farmers (29,006 cases, 59.86%), workers (5,194 cases, 10.72%), and retired individuals (4,722 cases, 9.74%; Figure 4).

### 3.3. Region distribution

**Figure 5:**
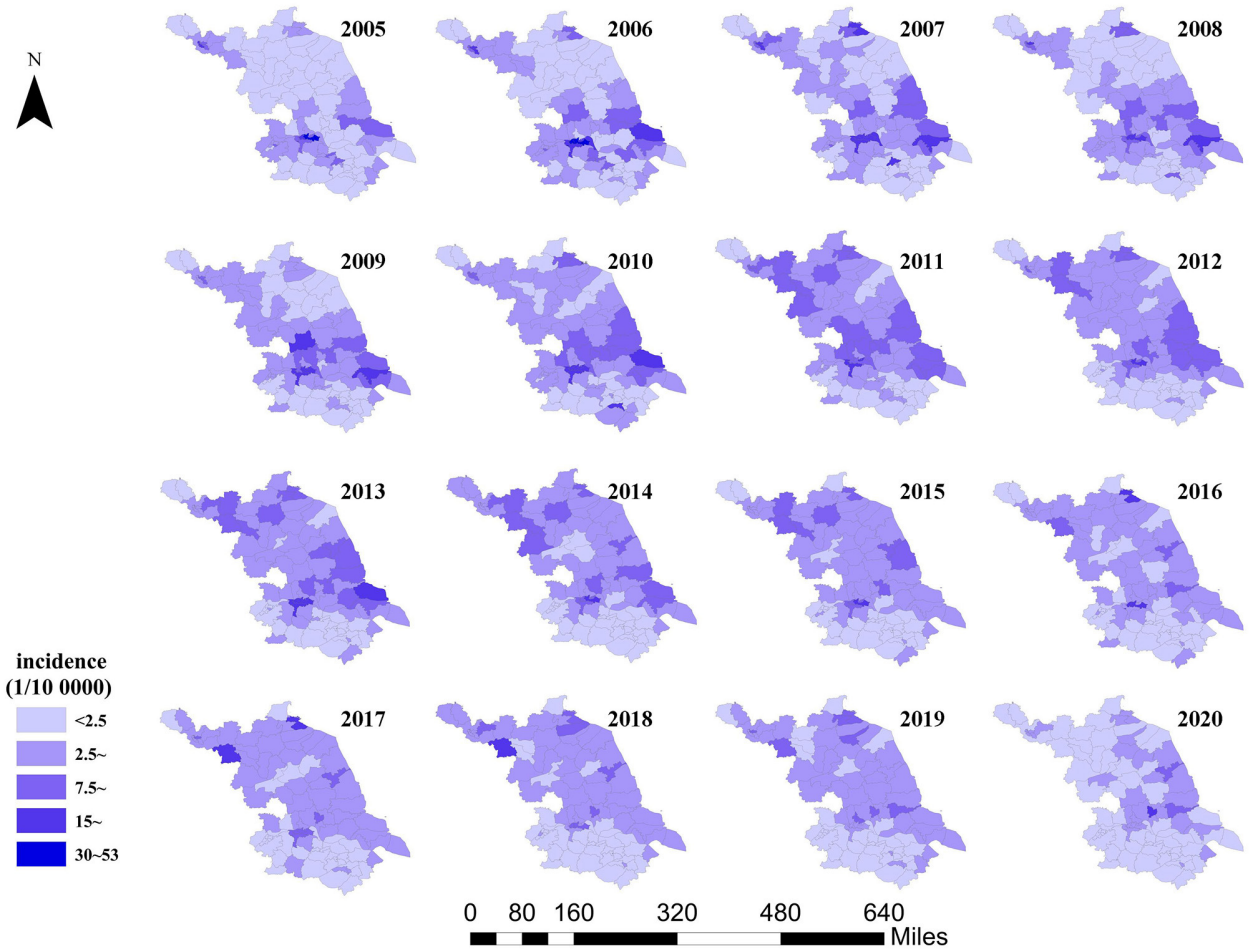
Distribution map of HE incidence in Jiangsu province from 2005 to 2020.

According to the distribution map of HE incidence in Jiangsu province from 2005 to 2020, the incidence in each county (city and district) shows dynamic changes over time, with obvious high-incidence and low-incidence areas. High-incidence areas are mainly located in Zhenjiang and Xuzhou cities, while low-incidence areas are located in Suzhou, Wuxi, and Changzhou cities. Both the high-incidence and low-incidence areas are clustered, showing a spatial clustering trend (Figure 5).

### 3.4. Spatial autocorrelation analysis

**Figure 6:**
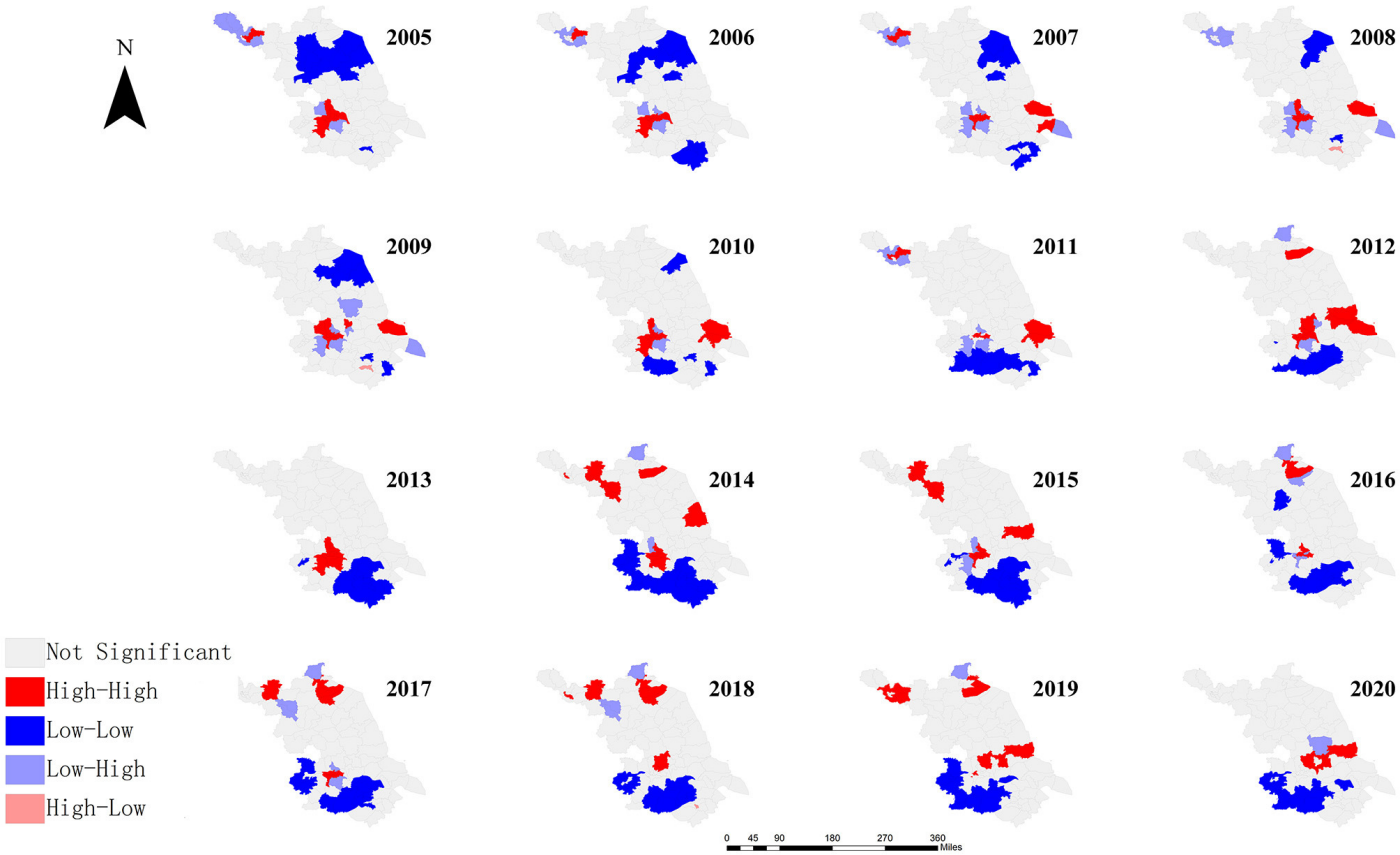
LISA of Jiangsu province from 2005 to 2020.

Global Moran's I of HE incidence in each year from 2005 to 2020 in Jiangsu province showed a positive value, ranging from 0.232 to 0.513, with *P*-values < 0.05 (Supplementary Table 1). Counties (cities and districts) with high HE incidence are adjacent. From 2005 to 2020, 31 counties (cities and districts) had high and statistically significant HE incidence. These counties are mainly located in Xuzhou and Zhenjiang cities (Figure 6).

### 3.5. Spatial-temporal scanning analysis

**Figure 7:**
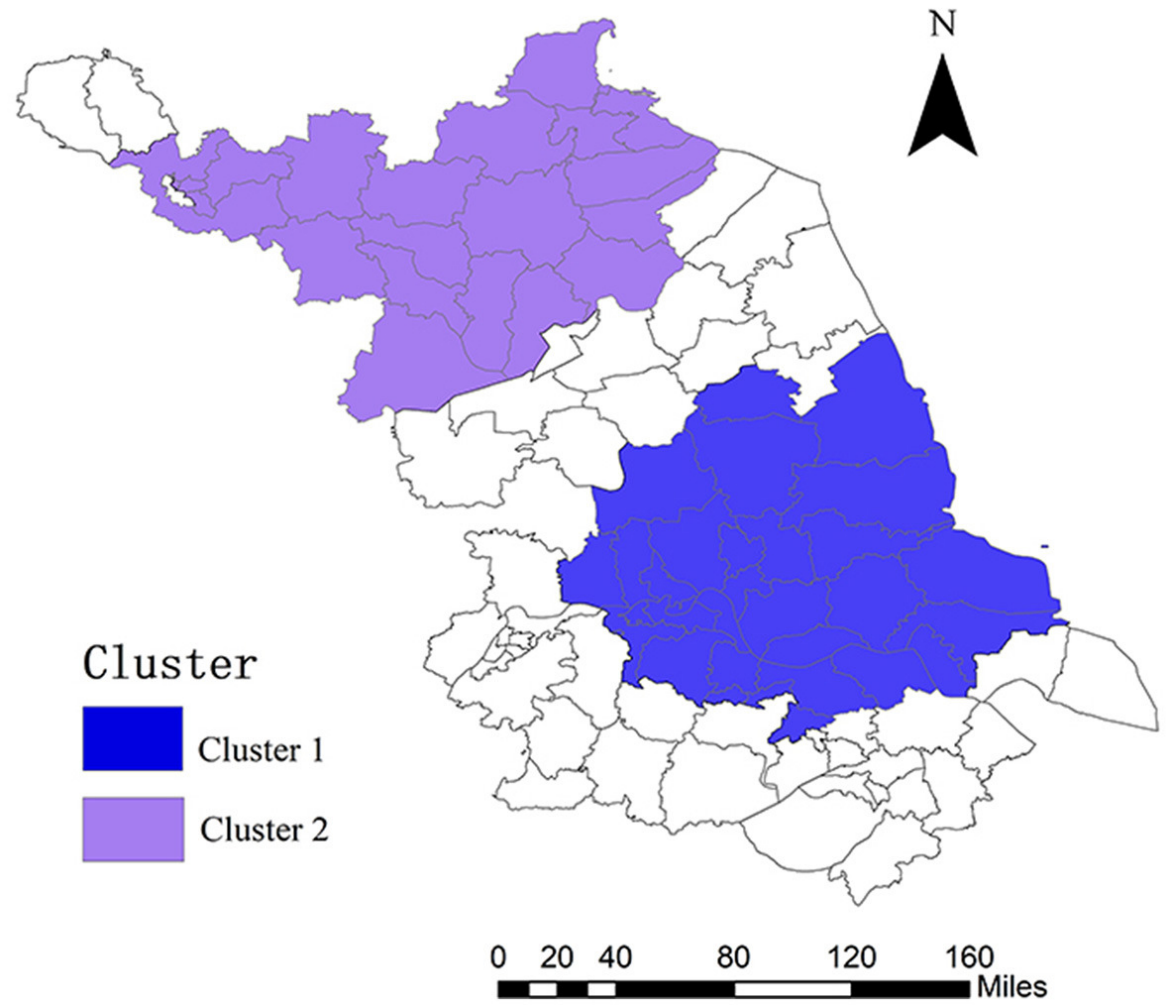
Spatial-temporal clustering of county-level HE cases in Jiangsu province from 2005 to 2020.

The spatial-temporal scanning analysis of HE cases in Jiangsu province from 2005 to 2020 identified two spatial clusters. A primary cluster is centered in Jiangyan District, with a radius of 98.60 km, and includes 30 counties (cities and districts), with a clustering time of 2006–2013 (RR = 2.02, LLR = 2,053.98, *P* < 0.001). A secondary cluster is centered in Xinyi City, with a radius of 106.56 km, and includes 20 counties (cities and districts), with a clustering time of 2011–2018 (RR = 1.70, LLR = 863.68, *P* < 0.001; Figure 7, Supplementary Table 2).

## 4. Discussion

Viral hepatitis is a key infectious disease in China (24), with one of the highest reported incidence rates among category B infectious diseases (25). In recent years, the incidence of hepatitis A has decreased year by year as China's national economy develops and people's living standards improve, especially with the availability of hepatitis A vaccines (26). In contrast, the incidence of HE in China has shown a significant upward trend, with the reported number of deaths exceeding that from hepatitis A every year (27). This situation poses a serious threat to the health and life safety of Chinese people. Therefore, the prevention and control of HE have become one of the major public health issues (28).

The results of this study show that from 2005 to 2020, 48,456 HE cases were reported in Jiangsu province, with an annual incidence rate of 3.87/100,000, higher than the levels in other provinces and municipalities such as Zhejiang, Shanghai, Anhui, and Hunan (3). This may be because Jiangsu is a coastal province where the tourism and catering industries are flourishing, and the population mobility is high (29) and may be related to the local dietary habits (30). The reported incidence showed an upward trend and then a downward trend, which is consistent with the trends of HE incidence reported in other provinces in China (21, 31). In 2020, however, the reported incidence decreased significantly, possibly due to the measures taken to control the COVID-19 pandemic that led to a reduction in social activities.

This study found that the incidence of HE has a significant seasonal pattern, with most cases reported in spring, especially in March, which is consistent with previous studies (32). This may be because of the higher frequency of large-scale population mobility and direct contact between people during the Spring Festival period, coupled with more opportunities for eating poultry, livestock (such as pigs), and seafood, which increase the risk of infection (14). At the same time, people have more social activities during holidays, and fatigue from long-distance travel may lead to a decrease in immunity and an increased susceptibility to infection (33). In addition, studies have shown that the incidence of HE is related to temperature, and a decline in temperature can lead to an increase in reported cases (34). The temperature in various regions of Jiangsu province was relatively low in March, which may be one of the reasons for the high number of reported cases in March.

The results of the study show that the incidence of HE is higher in males than in females and higher in middle-aged and older adult people than in other age groups. The HE patients are mainly farmers, which is consistent with the results in other regions outside Jiangsu province (35, 36). Possible reasons for this may be exposure opportunities, immunity, and different infection spectra in different age groups. Compared with other groups of people, adult males are engaged in more social activities, eat more frequently, and may have more exposure opportunities (37). Middle-aged and older adult people have poorer immunity and are more susceptible to illness after being infected. Infection usually presents apparent infection in adults and subclinical or silent infection in children and adolescents (38). HE in Jiangsu province is mainly a zoonotic disease. Hua et al. (39) found that there is 85–100% homology between the nucleotide sequence of 387 human HE cases and that of 5,588 pig HEV-4. The high percentage of farmers in reported cases may be related to the low awareness of hygiene and disease prevention in rural areas and poor living habits and environmental conditions. Other reasons include the risk of exposure increased by pigs and other livestock raised by farmers that may serve as reservoir hosts of the virus and patients' failure to seek timely medical treatment after the onset of the disease.

Spatial epidemiology is widely used in the surveillance of infectious diseases, particularly for analyzing the spatial distribution patterns and regional clustering of infectious diseases (40). Specifically, whether a disease outbreak has occurred in which region can be quickly identified by analyzing the spatial autocorrelation of infectious diseases to identify spatial clusters, which can provide a scientific basis for the formulation of prevention and control measures for infectious diseases (41).

HE showed a correlation at the provincial level and its overall incidence exhibited a spatial clustering pattern (20). Moran's I values of global spatial autocorrelation of HE incidence in different years were relatively stable, though different, and all positively correlated (*p* < 0.01). This suggests that areas with high HE incidence in Jiangsu province are surrounded by areas with similarly high incidence, while areas with low incidence are surrounded by areas with similarly low incidence, showing patterns of H-H and L-L clustering. Global Moran's I of HE incidence showed a trend of first increasing and then decreasing over time. The highest value was in 2014 (Moran's *I* = 0.513) and the lowest was in 2020 (Moran's *I* = 0.232), indicating that the correlation between HE incidence and spatial distribution in Jiangsu province showed a trend of increasing and then decreasing (42). The abovementioned results may be due to the fact that the hepatitis E vaccine was launched in October 2012, and the vaccine was promoted and used in Jiangsu province at the same time. With the improvement in living standards, people are paying more and more attention to the safety of water and food, and the supervision of food safety is increasing, which resulted in a continuous decline in the incidence of hepatitis E.

The results of local spatial autocorrelation analysis showed that H-H clustering occurred every year and the area and number of H-H clusters were different, mainly concentrated in areas such as Xuzhou, Lianyungang, Zhenjiang, and Nantong, involving 31 counties (cities and districts). Possible reasons are as follows: first, farmers in these areas largely raise livestock, which increases the risk of outdoor exposure; second, improvements in medical services have increased the detection rate; third, medical institutions have strengthened the quality of infectious disease reported, reducing the number of missed cases; fourth, the dietary habits of residents in coastal areas (i.e., frequently consuming seafood) increase the exposure risk.

To fully consider the relationship between the occurrence and development of HE in the temporal dimension, a spatial-temporal scan of the number of HE cases in Jiangsu province from 2005 to 2020 was performed. The results showed that the reported incidence of HE in the province from 2006 to 2018 showed clustering, with primary clusters from 2006 to 2010, both primary and secondary clusters from 2011 to 2013, and only secondary clusters from 2014 to 2018.

From 2006 to 2013, the center of the HE clustering area (primary clustering area) was in Jiangyan district, with a radius of 98.60 km, involving 30 counties (cities and districts), including Changzhou, Nantong, Suzhou, Taizhou, Wuxi, Yancheng, Yangzhou, and Zhenjiang. The risk of HE in this circular region was 2.021 times (RR = 2.021) that of other areas outside the clustering area.

From 2011 to 2018, the center of the HE clustering area (secondary clustering area) was in Xinyi city, with a radius of 106.564 km, involving 20 counties (cities and districts), including Xuzhou, Lianyungang, Suqian, and Huaian. Compared with other areas outside the clustering area, the risk of HEV in this circular region was 1.703 times higher (RR = 1.703). The risk of HE in this circular region was 1.703 times (RR = 1.703) that of other areas outside the clustering area.

The spatial-temporal scanning analysis indicated that the HE clustering area in Jiangsu province changed gradually from a primary clustering area to a secondary one from 2006 to 2018, suggesting that the overall transmission risk of HE in the province showed a downward trend. However, the clustering areas shifted from southern Jiangsu to northern Jiangsu, indicating a decline in the transmission risk of the disease in southern Jiangsu and rising risk in northern Jiangsu from 2006 to 2018.

However, this study has some limitations. First, only HE cases reported in the system were used for statistical analysis, and the data on some potential carriers or asymptomatic infected persons were not obtained, which may lead to selection bias. Second, this study covers a large period (covering reported cases of 16 years), during which some counties (cities and districts) may have had administrative merges or reorganizations. Therefore, the results may have been interfered with by the fluctuation of populations in different regions. Finally, this study did not collect and analyze the potential environmental factors, such as local customs and pig market distribution, that may affect the distribution of HE incidence.

## 5. Conclusion

In conclusion, examining the epidemiological characteristics, spatial distribution, and spatial-temporal clustering of HE cases in the counties (cities and districts) of Jiangsu province from 2005 to 2020 has significant public health implications for the effective prevention and control of the disease. It can also provide an important basis for the formulation of targeted prevention and control measures and in-depth research in areas with a high incidence of disease. This study is also feasible if real-time analysis is conducted. Data need to be analyzed daily, which requires more time and effort for statistical analysis.

## Data availability statement

The raw data supporting the conclusions of this article will be made available by the authors, without undue reservation.

## Ethics statement

This effort of disease control was part of the CDC's routine responsibility in Jiangsu province, China. Therefore, institutional review and informed consent were not required for this study. All data analyzed were anonymized.

## Author contributions

YS and WL designed research. XZ, YS, and XC analyzed data. YS and CB conducted the research and analyzed the results. YS, WL, and XC wrote the manuscript. All authors read and approved the final manuscript.
