# Supplementary material for: Analysis of the spatial-temporal distribution characteristics of hepatitis E in Jiangsu province from 2005 to 2020
Source: Front Public Health. 2023 Aug 8;11:1225261. doi: 10.3389/fpubh.2023.1225261 (PMC10442811; doi:10.3389/fpubh.2023.1225261)
Supplement: Supplementary file 1 [file Table_1.DOC]

| **Table 1 The Moran’s I of global spatial autocorrelation analysis during 2005 to 2020** | | | | |
| --- | --- | --- | --- | --- |
| **Year** | **Moran's I** | **Sd** | **Z-value** | **P-value** |
| 2005 | 0.407 | 0.060 | 6.931 | 0.001 |
| 2006 | 0.402 | 0.057 | 7.195 | 0.001 |
| 2007 | 0.303 | 0.065 | 4.854 | 0.001 |
| 2008 | 0.313 | 0.065 | 4.986 | 0.001 |
| 2009 | 0.420 | 0.065 | 6.730 | 0.001 |
| 2010 | 0.307 | 0.065 | 4.898 | 0.001 |
| 2011 | 0.422 | 0.066 | 6.621 | 0.001 |
| 2012 | 0.473 | 0.064 | 7.544 | 0.001 |
| 2013 | 0.512 | 0.063 | 8.378 | 0.001 |
| 2014 | 0.513 | 0.065 | 8.109 | 0.001 |
| 2015 | 0.454 | 0.065 | 7.207 | 0.001 |
| 2016 | 0.282 | 0.061 | 4.756 | 0.001 |
| 2017 | 0.331 | 0.065 | 5.294 | 0.001 |
| 2018 | 0.351 | 0.064 | 5.701 | 0.001 |
| 2019 | 0.375 | 0.065 | 5.897 | 0.001 |
| 2020 | 0.232 | 0.060 | 4.061 | 0.001 |
