# Supplementary material for: Analysis of the spatial-temporal distribution characteristics of hepatitis E in Jiangsu province from 2005 to 2020
Source: Front Public Health. 2023 Aug 8;11:1225261. doi: 10.3389/fpubh.2023.1225261 (PMC10442811; doi:10.3389/fpubh.2023.1225261)
Supplement: Supplementary file 2 [file Table_2.DOC]

**Table 2 Spatio-temporal scanning results of hepatitis E incidence in counties (districts, cities)of Jiangsu Province from 2005 to 2020**

| **Cluster** | **Counties (Districts, Cities)** | **Radius**  **(km)** | **Time** | ***RR*** | ***LLR*** | ***P* value** |
| --- | --- | --- | --- | --- | --- | --- |
| NO.1 | Jingkou District, Runzhou District, Dantu District, Danyang City, Yangzhong City, Guangling District, Hanjiang District, Jiangdu District, Yizheng City, Gaoyou City, Yandu District, Dafeng District, Dongtai City, Huishan District, Jiangyin City, Hai Ling District, Gaogang District, Jiangyan District, Xinghua City, Jingjiang City, Taixing City, Chongchuan District, Tongzhou District, Rudong County, Rugao City, Haian City, Tianning District, Zhonglou District, Xinbei District, Zhangjiagang City | 98.599 | 2006-2013 | 2.021 | 2053.983 | <0.001 |
| NO.2 | Gulou District, Yunlong District, Jiawang District, Suining County, Xinyi City, Pizhou City, Sucheng District, Suyu District, Shuyang County, Siyang County, Sihong County, Lianyun District, Haizhou District, Ganyu District, Donghai County, Guanyun County, Guannan County, Huaiyin District, Lianshui County, Tongshan District | 106.564 | 2011-2018 | 1.703 | 863.682 | <0.001 |
